# Activity of tannins from *Stryphnodendron adstringens *on *Cryptococcus neoformans*: effects on growth, capsule size and pigmentation

**DOI:** 10.1186/1476-0711-8-29

**Published:** 2009-11-05

**Authors:** Kelly Ishida, Sonia Rozental, João Carlos Palazzo de Mello, Celso Vataru Nakamura

**Affiliations:** 1 Laboratório de Biologia Celular de Fungos, Instituto de Biofísica Carlos Chagas Filho, Universidade Federal do Rio de Janeiro, Rio de Janeiro/RJ, Brazil; 2 Departamento de Farmácia e Farmacologia, Universidade Estadual de Maringá/PR, Brazil; 3 Laboratório de Microbiologia Aplicada a Produtos Naturais e Sintéticos, Universidade Estadual de Maringá/PR, Brazil

## Abstract

**Background:**

*Stryphnodendron adstringens *(Mart.) Coville, Leguminosae, also known in Brazil as barbatimão, is rich in tannins and many flavan-3-ols and proanthocyanidins such as prodelphinidins and prorobinetinidins. Previous studies have demonstrated several pharmacological properties of tannins from barbatimão, including anti-candidal activity.

**Methods:**

The antifungal activity of proanthocyanidin polymeric tannins from *Stryphnodendron adstringens *(subfraction F2.4) was evaluated against three strains of *Cryptococcus neoformans *with different capsule expressions, using the broth microdilution technique, light microscopy and transmission electron microscopy. The effect of subfraction F2.4 on *C. neoformans *and melanoma mammalian cells pigmentation was also evaluated.

**Results:**

Although susceptibility assays revealed MIC values quite similar (between 2.5 and 5.0 μg/ml), analyses of MFC values revealing that the acapsular mutant Cap 67 was more susceptible to be killed by the subfraction F2.4 (MFC = 20 μg/ml) than the two tested capsular strains (T_1_-444 and ATCC 28957) (MFC > 160 μg/ml). Optical and electron microscopy experiments revealed relevant alterations in cell shape and size in all strains treated with 1 and 2.5 μg/ml of subfraction F2.4. Capsule size of the capsular strains decreased drastically after subfraction F2.4 treatment. In addition, ultrastructural alterations such as cell wall disruption, cytoplasm extraction, mitochondria swelling, increase in the number of cytoplasmic vacuoles and formation of membranous structures in the cytoplasm were also observed in treated yeasts. Incubation with subfraction F2.4 also decreased *C. neoformans *pigmentation, however, did not interfere in melanization of B_16_F_10 _mammalian cells.

**Conclusion:**

Our data indicate that tannins extracted from *S. adstringens *interfered with growth, capsule size and pigmentation, all important virulence factors of *C. neoformans*, and may be considered as a putative candidate for the development of new antifungal agents.

## Background

*Stryphnodendron adstringens *(Mart.) Coville, Leguminosae, also known in Brazil as barbatimão, is used in the form of a decoction or infusion as an adstringent, anti-diarrhoeal, antimicrobial and hypoglycaemic agent for the treatment of gynaecological problems and healing wounds [1]. The stem bark of barbatimão is rich in tannins (10-37%) [2], many flavan-3-ols and proanthocyanidins such as prodelphinidins and prorobinetinidins [3,4]. Previous studies have demonstrated cicatrizing properties [5], analgesic and anti-inflammatory activity [6,7] and gastric anti-ulcerogenic effects [8] of the tannins extracted from the stem bark of barbatimão.

Also the anti-protozoal effect of tannins from barbatimão has been described against *Herpetomonas samuelpessoai *[9], *Trypanosoma cruzi *and *Leishmania amazonensis *[10,11] and antiviral activity against bovine herpesvirus and poliovirus were also demonstrated by Felipe *et al*. [12]. In addition, our group showed a high antifungal activity of a polymeric tannin (hexameric compound), composed of monomeric units of prodelphinidins and prorobinetinidins, from the stem bark of barbatimão, against clinical isolates of *Candida albicans *[13].

*Cryptococcus neoformans *is an encapsulate opportunistic yeast that can cause cryptococcosis, predominantly in immunocompromised patients with underlying predisposing factors, such as organ transplantation, haematological malignancies and advanced human immunodeficiency virus diseases [14]. This yeast is ubiquitous in the environment and is acquired by inhalation of desiccated particles, causing pulmonary cryptococcosis that may be accompanied by systemic dissemination and it usually manifests itself as meningoencephalitis [14]. The choice of treatment for cryptococcosis depends on the anatomic sites of involvement and the host's immune status. Treatment is usually based on amphotericin B therapy with or without flucytosine and azole agents. Fluconazole and itraconazole are the drugs of choice for prophylaxis and maintenance therapy [15]. Although resistance to antifungal drugs is rare in *C. neoformans*, long-term suppressive regimens are raising concern about the development of drug resistance [16]. In addition to the limited number of available therapeutic options to treat cryptococcal infections, prolonged therapy may increase the toxic effects of these drugs on the patient.

*C. neoformans *is unique among pathogenic fungi in having a polysaccharide capsule, mainly composed of glucuronoxylomannan (GXM) and galactoxylomannan (GalXM), which is considered the major contributor to its virulence. GXM and GalXM units can be released during cryptococcal infection with deleterious effects to the host immune response, such as anti-phagocytic and immunosuppressive capacity [17].

Melanin synthesis during infection is also considered an important factor in the virulence of *Cryptococcus*. Melanin protects from oxygen and nitrogen oxidants, microbicidal peptide activity, ingestion and killing by macrophages. In addition, melanized *C. neoformans *are less susceptible to antifungal agents [18].

Drugs interfering in the growth and virulence factors of *C. neoformans *can be considered stronger candidates for the study and development of new antifungal agents. The aim of this study was to evaluate the antifungal activity of the polymeric tannin (hexameric compound) extracted from stem bark of *S. adstringens *against *C. neoformans *strains and to observe its effect on cell growth, morphology, ultrastructure, capsule size and pigmentation.

## Methods

### Extraction and characterization of subfraction F2.4 from *S. adstringens*

The stem bark of *S. adstringens *was collected, dried and powdered. The crude extract, fractions and subfractions (including F2.4) were obtained as described by Ishida *et al*. [13]. Briefly, the crude extract was obtained by turbo-extraction (Skymsen) of 100 g of the bark with 70% acetone in water for 20 min. The organic solvent was eliminated by reduced pressure and lyophilized to yield a crude extract (F1). Next, the F1 (36 g) was suspended in water (360 mL) and partitioned with ethyl acetate (360 mL) to obtain a water fraction (F2) and an ethyl acetate fraction (F3). The F2 fraction (2 g) was chromatographed on a Sephadex_LH-20 column (h = 170 mm; j = 21 mm, Pharmacia), using one sequence of eluent system of volumetric proportions with water (50% ethanol, 70% ethanol, 90% ethanol and 70% acetone), obtaining four subfractions. Chemical characterization of subfraction F2.4 was analysed by mass spectrometry ES-MS and ^13^C NMR spectroscopy and characterized as a proanthocyanidin polymer (a hexameric compound), composed of prodelphinidin and prorobinetinidin units and gallic-acid residues, with an average molecular weight of 2,114 Da. For the experiments realized in this work, the subfraction F2.4 lyophilized powder (maintained at -20°C freezer) was diluted directly in RPMI 1640 medium.

### Microorganisms

Strains of *C. neoformans *with different capsular expressions were used in this study. *C. neoformans *T_1_-444 (serotype A, capsular size around 2.85 μm) is a clinical isolate from a patient with meningoencephalitis and AIDS, provided by the Hospital of São Paulo Federal University. The other two strains (ATCC 28957 - isolated from human bone lesion - capsular size around 2.66 μm) and Cap 67 acapsular mutant [19] were purchased from the American Type Culture Collection (ATCC). Cap 67 acapsular mutant is deposited in the American Type Culture Collection as ATCC 52817. Stock cultures were maintained on Sabouraud dextrose agar, at 4°C. Subcultures were made for each experiment in the same medium, at 35°C, for 72 h.

### Culture cells

B_16_F_10 _murine melanoma cells were kindly provided by Dr. J. Morgado-Díaz (Instituto Nacional de Cancer, Divisão de Biologia Celular, Rio de Janeiro/Brazil) and maintained in RPMI 1640 medium (Gibco Invitrogen Corporation, New York, USA), supplemented with 2 mM L-glutamine and heat-inactivated 10% foetal bovine serum, buffered with sodium bicarbonate and 50 μg/ml gentamicin. The cultures were maintained at 37°C, in 5% CO_2_.

### Anti-cryptococcal activity of subfraction F2.4

Minimum inhibitory concentration (MIC) determination was performed as described in document M27-A3 [20]. Briefly, a two-fold serial dilution of subfraction F2.4 in RPMI 1640 medium without sodium bicarbonate (Sigma Chemical Co., MO, USA) buffered with 0.165 M MOPS (Sigma Chemical Co., MO, USA) was made in 96-well microtitre trays, to obtain concentrations of 0.31 to 160 μg/ml. A suspension of *C. neoformans *of 1-5 × 10^6 ^cfu/ml was prepared, diluted 1:1000 and 100 μl was dispensed into each well containing 100 μl of medium to obtain a final concentration of 0.5-2.5 × 10^3 ^cfu/ml. The microtitre trays were incubated at 35°C for 72 h in a humid chamber. The MIC values were considered to be the lowest concentration that visibly inhibited cryptococcal growth. Fluconazole (Pfizer, São Paulo, Brazil), itraconazole (Sigma Chemical Co., MO, USA), and amphotericin B (Sigma Chemical Co., MO, USA) were used as standard antifungal.

Minimum fungicidal concentration (MFC) was determined using an aliquot of 10 μl of the yeast suspension treated with inhibitory concentrations of subfraction F2.4 and subcultured on a drug-free Sabouraud dextrose agar, incubated at 35°C, for 72 h. The MFC was determined as the lowest concentration that showed negative culture [13].

### Light microscopy

Strains of *C. neoformans *were treated with 1 and 2.5 μg/ml of subfraction F2.4 in RPMI 1640 medium buffered with 0.165 M MOPS, for 72 h, at 35°C. The yeasts were collected, washed in PBS, pH 7.2 and negatively stained with India ink. The images were observed by differential interference contrast (DIC) microscopy with an Axioplan 2 (Zeiss, Germany) optical microscope, acquired using a Color View SX digital camera and processed with the program analysis (Soft Image System, Germany). Morphological alterations and budding cells were counted in at least 300 yeasts. The cell and capsule size were measured by the SemAfore 5.0 program (Jeol, Japan).

### Transmission electron microscopy

Strains of *C. neoformans *were treated with 1 and 2.5 μg/ml of subfraction F2.4 for 72 h, at 35°C. The yeasts were collected, washed in PBS, pH 7.2 and then fixed in 2.5% glutaraldehyde and 4% paraformaldehyde, in 0.1 M cacodylate buffer. They were then post-fixed in 1% osmium tetroxide in cacodylate buffer containing 1.25% potassium ferrocyanide and 5 mM CaCl_2 _for 2 h, serially dehydrated in ethanol and embedded in Spurr epoxy resin. Ultrathin sections were obtained on a Reichert Ultracut, stained with 5% uranyl acetate and 0.5% lead citrate and observed in a Zeiss CEM-900 electron microscope.

### Effect of subfraction F2.4 on cryptococcal pigmentation

Minimum agar medium (15 mM dextrose, 10 mM MgSO_4_, 29.4 mM KH_2_PO_4_, 13 mM glycine, 3 μM thiamine-HCl and 2% agar) supplemented with 1 mM L-3,4-dihydroxyphenylalanine (L-dopa, Sigma Chemical Co., MO, USA) was used to induce melanization of *C. neoformans*. Subfraction F2.4 was diluted in minimum agar medium to obtain concentrations of 5 to 100 μg/ml. An aliquot of 20 μL of 1 × 10^6 ^cfu/ml of the *C. neoformans *suspension was dispensed on the medium and then incubated in a dark humid chamber for 5 days, at 35°C. Medium without L-dopa was used as a negative control for melanin synthesis. Medium - without yeast - supplemented with L-dopa was used to observe if autopolymerization occurred.

The images were obtained using a transilluminator and a digital camera without flash illumination. The colony pigmentation was measured using the program Image J, developed by the National Institutes of Health (NIH, USA) . The percentage of pigmentation inhibition was calculated by comparison with the positive control for cryptococcal pigmentation.

### Effect of subfraction F2.4 on B_16_F_10 _cell melanogenesis

A B_16_F_10 _cell suspension of 2.5 × 10^5 ^cell/ml was dispensed into each well of 6-well microplates, for 24 h, to allow the cells to adhere. Subsequently, the cells were treated with several concentrations of subfraction F2.4 (1, 10, 50, and 100 μg/ml) for 48 h, at 37°C and 5% CO_2_. The cell monolayers were resuspended in PBS, pH 7.2, and counted in a haemocytometer chamber. Cell suspension was treated with 1 N NaOH for 24 h, at 37°C, for melanin extraction. Absorbance was determined in a spectrophotometer (Model UV-1700-Pharmaspec, Shimadzu, Japan) at 475 nm wavelength. Melanin concentration was determined using a standard curve for synthetic melanin (Sigma Chemical Co., MO, USA). Synthetic melanin standard curve was constructed using 0.78, 1.56, 3.12, 6.25, 12.5, 25 and 50 μg/ml of melanin diluted in 1 N NaOH and the absorbance at 475 nm wavelength of each concentration was obtained with a quartz cuvette containing 1 ml of each solution. Background of the each concentration was subtracted using absorbance the 1 N NaOH solution. The melanin standard curve presented a linear profile (R^2 ^= 0.9999). Than, melanin concentration per 10^5 ^cells was calculated.

### Statistical analysis

The results were analyzed using the Prism 5.0 computer program. The data obtained in experimental models were evaluated by one-way analysis of variance (ANOVA) followed by Dunnett's test. Differences between the control raw mean and the drug groups raw means were considered to be statistically significant when p < 0.05.

## Results

### Anti-cryptococcal activity of polymeric tannin from *S. adstringens*

The MICs of subfraction F2.4 were 2.5 and 5 μg/ml to acapsular and capsular strains, respectively (Table 1). Additionally, sub-inhibitory concentrations, 1 and 2.5 μg/ml, of subfraction F2.4 inhibited the growth of ATCC 28957 strain by 16.67% and 36.67%, respectively and of Cap 67 strain by 56.87% and 86.87%; but did not inhibit the growth of T_1_-444 strain. Although the acapsular strain Cap 67 had shown similar susceptible to subfraction F2.4 as the T_1_-444 and ATCC 28957 strains, the MFC values of Cap 67 were at least 8 times lower when compared to the MFC values of the capsular strains (Table 1). T_1_-444, ATCC 28954 and Cap 67 strains were all susceptible to fluconazole, itraconazole and amphotericin B. We did not find any relevant differences between the MIC or MFC values of the three tested strains in relation to fluconazole, itraconazole and amphotericin B (Table 1).

**Table 1:** Anti-cryptococcal activity of subfraction F2.4 from *Stryphnodendron adstringens *against strains of *Cryptococcus neoformans *with different capsule expressions: T_1_-444 strain, ATCC 28957 strain and an acapsular mutant (Cap 67 strain).

**Drugs**	***C. neoformans*** **T**_**1**_**-444**		***C. neoformans*** **ATCC 28957**		***C. neoformans*** **Cap67**	
	
	**MIC**	**MFC**	**MIC**	**MFC**	**MIC**	**MFC**
Subfraction F2.4	5	>160	5	>160	2.5	20
Fluconazole	4	16	2	8	2	8
Itraconazole	0.12	8	0.12	8	0.03	8
Amphotericin B	0.12	0.25	0.06	0.25	0.06	0.12

Minimum inhibitory concentration (MIC) and minimum fungicidal concentration (MFC) are in μg/ml.

### Light microscopy

**Figure 1:**
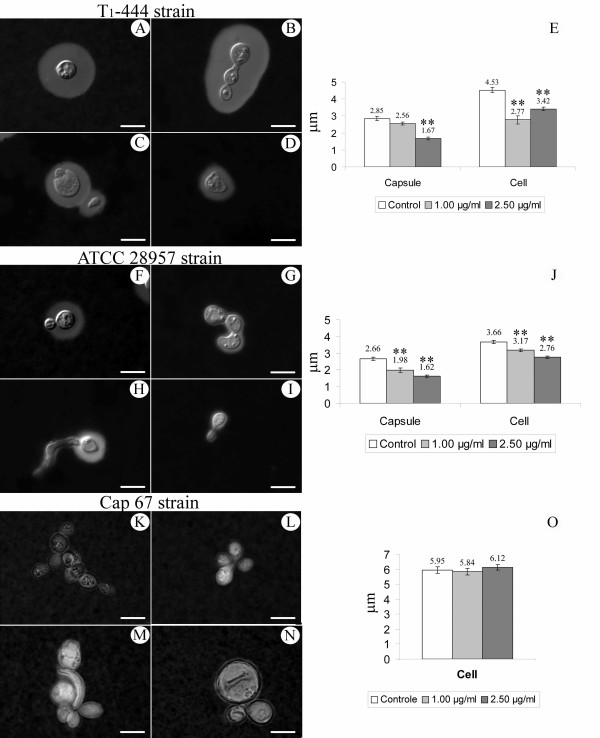
**Morphological alterations of *C. neoformans *strains (T_1_-444, ATCC 28957 and Cap 67 strains) treated with 1 and 2.5 μg/ml of subfraction F2.4 from *S. adstringens***. **A, F and K: **Control; **B, G and L: **1 μg/ml; **C-D, H-I and M-N: **2.5 μg/ml; **E, J and O: **capsule and cell size (μm) of *C. neoformans *strains untreated and treated with subfraction F2.4. ** p < 0.01.

In order to examine the effect of subfraction F2.4 on the morphology of capsular (ATCC 28954 and T_1_-444) and acapsular (CAP 67) strains of *C. neoformans*, the yeasts were cultivated or not (control cells) in the presence of the drug and examined by light microscopy. Control capsular cells showed a regular oval cell shape and are homogeneously surrounded by the polysaccharide capsule (Figs. 1A and 1F). The treatment of the strains with 1 and 2.5 μg/ml of subfraction F2.4 lead to a significant reduction in capsule size (Figs. 1E and 1J) and decrease of cell size when compared with the control cells (Figs. 1E and 1J).

Treatment of these yeasts with subfraction F2.4 also leads to several morphological alterations such as: (i) the non separation of young bud cells (Figs. 1B-C and 1G-H); (ii) elongation of the young buds (Fig. 1H); and (iii) appearance of smaller and anamorphous yeasts (Figs. 1D and 1I). The capsular strains of *C. neoformans *did not show any significant differences in the counting of the yeast budding.

The untreated yeasts of *C. neoformans *Cap 67 showed agglomerate yeasts characteristic of this strain (Fig. 1K). For this reason, it was not possible to count the yeast buds. No significant alteration in cell size was observed. However, we observed many changes in yeast morphology such as the appearance of decreasing moon (Fig. 1M) and of globular cells (Fig. 1N) after incubation with subfraction F2.4.

### Transmission electron microscopy

**Figure 2:**
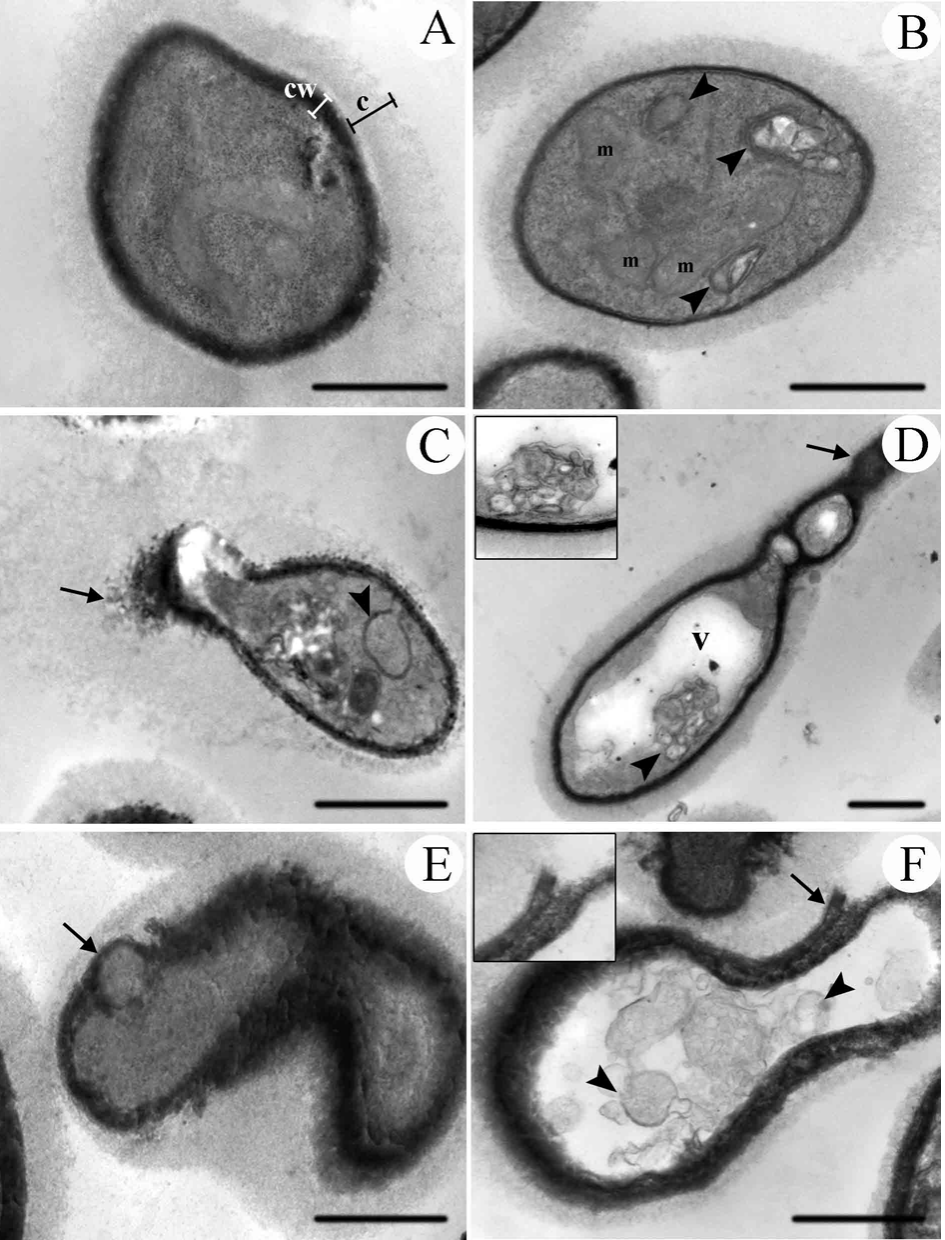
**Transmission electron micrographs of *C. neoformans *ATCC 28957 strain grown in the presence of subfraction F2.4 from *S. adstringens *for 72 h, at 35°C**. Untreated yeasts have a compact cell wall (cw) surrounded by a polysaccharide capsule (c) **(A); **Yeasts treated with 1 μg/ml **(B-D) **and 2.5 μg/ml **(E-F) **show several ultrastructural alterations, such as cell-wall disruption (black arrow), presence of vacuoles (v) mitochondrial swelling (m) and many membranous structures in the cytoplasm (black arrowhead). Bars = 1 μm.

Untreated yeasts had a compact cell wall (cw), capsule (c) and a cytoplasm with a regular electron density and many ribosomes (Fig. 2A). Ultrastructural analyses of *C. neoformans *ATCC 28957 revealed several morphological alterations after treatment with subfraction F2.4 (Figs. 2B-F). Yeast of *C. neoformans *ATCC 28957 treated with 1 μg/ml of the subfraction F2.4 showed disruption of the cell wall (arrow, in Figs. 2C-D), changes in the yeast morphology and in the budding process (Figs. 2C-D). The presence of membranous structures (arrowheads, in Figs. 2B-C), large vacuoles (v, in Fig. 2D), mitochondria swelling (m, in Fig. 2B) were found in the cytoplasm of the treated yeasts. Yeasts treated with 2.5 μg/ml of subfraction F2.4 showed more-drastic alterations with irregular buds emerging from the lateral of the mother cells instead of from the usual terminal, complete loss of the normal cellular ovular shape, cell wall detachment (arrows), membranous structures (arrowheads) and extracted cytoplasm suggesting cell death (Figs. 2E-F). The T_1_-444 strain showed similar alterations in cellular ultrastructure, however less pronounced than the ATCC 28957 strain (data not shown).

**Figure 3:**
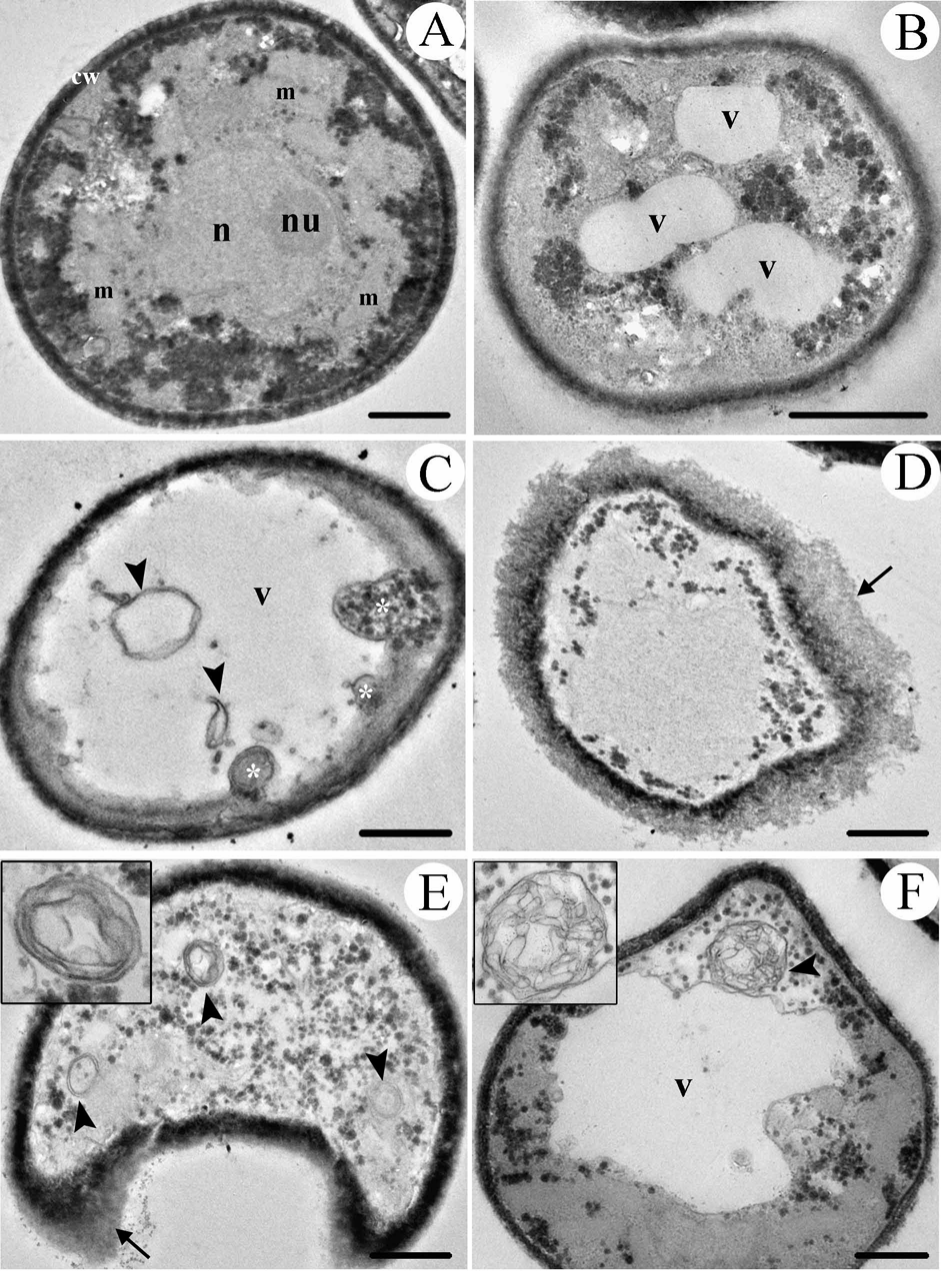
**Transmission electron micrographs of acapsular mutant Cap 67 grown in the presence of subfraction F2.4 from *S. adstringens *for 72 h, at 35°C**. Untreated yeasts have a compact cell wall (cw), nucleus (n), nucleolus (nu), mitochondria (m) and many electron-dense granules (glycogen) **(A)**. Yeasts treated with 1 μg/ml **(B-D) **and 2.5 μg/ml **(E-F) **showed drastic ultrastructural alterations. Disruption of cell wall (black arrow), membranous structures (black arrowhead and details in the box of Fig. E and F), vacuoles (v) and budding of vesicular bodies in the vacuole lumen (white asterisk) can be observed. Bars = 1 μm.

Yeasts of the acapsular mutant (Cap 67) presented more drastic alterations when grown in the presence of 1 μg/ml (Figs. 3B-D) or 2.5 μg/ml (Figs. 3E-F) of subfraction F2.4. The ultrastructure of untreated yeasts was preserved, with compact cell wall (cw), nucleus (n), nucleolus (nu), mitochondria (m) and electron-dense glycogen granules (Fig. 3A). In contrast, treated yeasts showed many ultrastructural alterations such as budding of vesicular bodies into vacuoles lumen (Fig. 3C, white asterisk), cell wall disassembling (arrows in Figs. 3D-E), an increase in number and in size of electron lucent vacuoles (v) (Figs. 3B-C and 3F) and intracellular membranous structures (arrowheads in Figs. 3E-F). These membranous structures were also observed inside the vacuoles (arrowheads in Fig. 3C).

### Polymeric tannin reduce cryptococcal pigmentation

**Figure 4:**
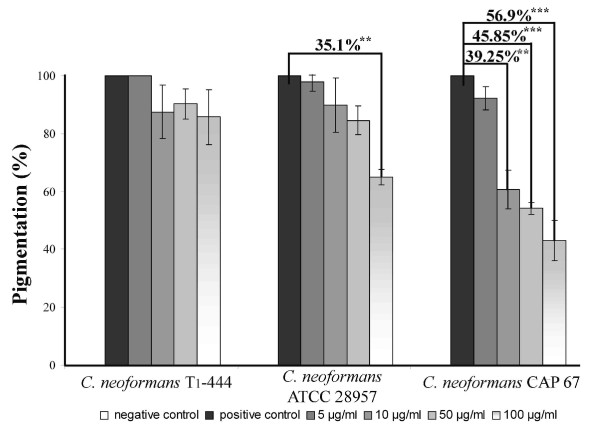
**Pigmentation percentage of *C. neoformans *strains treated with several concentrations of the subfraction F.24 (5 to 100 μg/ml) in minimum medium supplemented with 1 mM L-dopa**. Subfraction F2.4 from *S. adstringens *reduced the pigmentation of *C. neoformans *with different capsule expressions. **p < 0.01; *** p < 0.001.

*C. neoformans *treated with subfraction F2.4 grown in minimum agar medium supplemented with 1 mM L-dopa were significantly less pigmented than the control cells (Fig. 4). The hexameric condensed tannin was more active to inhibit the pigmentation of the acapsular strain Cap 67 than the capsular strains. Pigmentation was strongly reduced in Cap 67 at concentrations of 10-100 μg/ml (39.25-56.9%, p < 0.01), however in ATCC 28957 it was significantly reduced only in 100 μg/ml of subfraction F2.4 (35.1%, p < 0.01). The polymeric tannin did not affect T_1_-444 strain pigmentation at concentrations ≤100 μg/ml.

### Effect of the polymeric tannin on melanogenesis of B_16_F_10 _cells

The effect of subfraction F2.4 on mammalian cell (B_16_F_10_) melanogenesis was also evaluated. Concentrations ≤100 μg/ml of subfraction F2.4 did not alter melanin synthesis in B_16_F_10 _cells (p > 0.05) (data not shown).

## Discussion

The small number of commercial antifungal agents, the inappropriate pharmacokinetics and the toxic effects are important factors related to unsuccessful treatments of cryptococcosis and other mycosis. Consequently there is an increasing need for new compounds with antifungal activity. Natural products, including plants, may be a source of compounds with antifungal effects and therefore possible candidates for the development of new antifungal agents [21,22].

In this study, we observed the activity of subfraction F2.4 (a hexameric compound) extracted from the stem bark of barbatimão, against three strains of *C. neoformans *with different capsule expressions. The acapsular mutant Cap 67 was more susceptible than the capsular strains; although only small differences were observed between MIC values of the three strains the MFC results for the capsulate strains was at least 8 times higher than for the acapsular one. These data suggest that the presence of a capsule surrounding *C. neoformans *cells may influence the anti-cryptococcal activity of the hexameric compound (Table 1). Interestingly, the presence of a capsule did not interfere with the action of fluconazole, itraconazole or amphotericin B.

Yeasts treated with sub-inhibitory concentrations of subfraction F2.4 showed changes in cell morphology and also their cell size and capsule size decreased significantly when compared to untreated yeasts. The treatment with hexameric compound may interfere with the uptake of nutrients important for normal fungal development.

Several ultrastructural alterations of *C. neoformans *treated with subfraction F2.4 were observed including the presence of yeasts with altered shape, an amorphous material shading from the cell wall, an increase in the size of vacuoles, appearance of membranous structures and mitochondria swelling. Similar mitochondrial alterations were previously reported by Holetz et al. after treatment of protozoan by tannins [9]. These authors suggested the involvement of the tannins in the oxidative phosphorylation and electron transport defects. Interestingly, yeasts of the acapsular mutant *C. neoformans *Cap 67 showed more drastic ultrastructural alterations than the other strains.

The formation and budding of vesicles in the vacuoles lumen can be characterized as autophagic bodies, suggesting death of *C. neoformans *by autophagy [23]. In addition, the structures of concentric membranes are similar to myelin-like figures, which are also related to an autophagic process [24]. These structures were more frequently observed in the treated Cap 67 strain. Although autophagy is a cell mechanism that is necessary for the survival and pathogenesis of many fungi, including *C. neoformans *[25,26], it is also a mechanism of fungal death induced by antifungal drugs [27-29].

In a previous study, we demonstrated antifungal activity of subfraction F2.4 extracted from barbatimão against *C. albicans *isolates [13]. We observed growth inhibition and alteration of the cell wall of *C. albicans*, which may be related to a change in cell surface hydrophobicity, decrease in the capacity of adherence to eukaryotic cells and glass surfaces, inhibition of germ-tube formation and stimulus of phagocytosis by macrophages. The anti-candidal activity was related to the polymeric tannin present in subfraction F2.4 (hexameric compound), which is composed of monomeric units of proanthocyanidins (prodelphinidins and prorobinetinidins) and gallic-acid residues.

A wide range of antimicrobial activity (against filamentous fungi, yeasts and bacteria), stimulation of phagocyte cells and host-mediated tumour activity have been assigned to tannins [30]. Polyphenols may precipitate and/or complex with a variety of macromolecules, including polysaccharides, proteins, alkaloids, polymers and cyclodextrins [31]. They appear to associate with macromolecules through non-specific forces such as hydrogen bonding to H-bond-accepting groups and hydrophobic interactions arising from the aromatic rings in their gallic acids, as well as by covalent-bond formation [31]. The increase in tannin polymerisation also increases the degree of intermolecular reaction [32]. In addition, the number of hydroxyl groups on the B-ring affects the level of growth inhibition of many microorganisms [33], suggesting that the trihydroxylated B-rings of proanthocyanidins may have higher antimicrobial action [13]. Thus, the antifungal activity of subfraction F2.4 polymeric tannin against *C. neoformans *strains may be related to the ability of tannins to inhibit extracellular microbial enzymes, deprivation of substrates and metal-ion cell-envelope transport proteins and the direct action on microbial metabolism through inhibition of oxidative phosphorylation [33].

The capsular compounds GXM and GalXM are synthesized intracellularly, transported to the extracellular space inside membrane vesicles and then released extracellularly for capsular enlargement. However, it is not clear how GXM and GalXM fibres are incorporated into the capsule [34]. It has been suggested that capsular assembly in *C. neoformans *results from divalent cation-mediated self-aggregation of extracellular-accumulated GXM molecules [35]. Interestingly, the hexameric compound of subfraction F2.4 promoted a reduction of the capsule size and this effect seemed to be dose-dependent. He *et al*. [36] showed that hydrogen bonding is the predominant effect in the interactions between gallotannins and carbohydrates. Subfraction F2.4 polymeric tannin could act by inhibiting the incorporation of capsular components by hydrogen bonding or divalent-cation scavenging. However, we observed that anti-cryptococcal activity of subfraction F2.4 previously incubated with the supernatant of *C. neoformans *T_1_-444 strain culture (rich in capsular components) was similar to that of subfraction F2.4 alone (data not shown). Together, these data suggest that the hexameric compound of subfraction F2.4 is not inhibiting the incorporation of capsular components but is probably interfering in other processes, such as the synthesis of capsular compounds and/or the transport of vesicles to the extracellular moiety.

Melanin is a pigment produced by several pathogenic fungi and is considered to be an important virulence factor for *C. neoformans *[18]. Its synthesis depends on laccase and also on the presence of exogenous substrates (such as L-dopa and epinephrine). Laccase is a phenoloxidase enzyme present in the cell wall that possesses a broad spectrum of activity, oxidizing polyphenolic compounds. Although the details of the chemical structure of melanin are largely unknown, it is believed to be a cross-linked polymer of phenol and indole subunits that are organized in many spherical granular particles arranged in multiple concentric layers in the cell wall [37]. Recently, Rodrigues *et al*. [38] demonstrated the secretion of pathogenesis-related molecules, including a laccase, by trans-cell-wall transport into membrane vesicles.

Baurin *et al*. [39] showed that the extract from stem bark of barbatimão strongly inhibits (close to 90%) *Neurospora crassa *tyrosinase, a phenoloxidase enzyme. We suggest that the hexameric compound of subfraction F2.4 could inhibit the synthesis of melanin by inhibiting laccase enzyme or by inhibiting cross linking of phenol and indole units. Analyzing the protein alignment of human tyrosinase (E.C.1.14.18.1) and *C. neoformans *laccase (E.C.10.32), both phenoloxidase enzymes, we can observe low similarity comparing both aminoacid sequences, using the NCBI BLAST databases at National Center for Biotechnology Information (NCBI - ) (data not show). These data may explain the possible selectivity of the hexameric compound for *C. neoformans *laccase enzyme. In addition, interference in the nutrient acquisition and changes in the cell wall morphology caused by the treatment with hexameric compound of subfraction F2.4 could also be interfering with the melanin synthesis and its destination. Disturbance of the homeostasis of melanin synthesis by the hexameric compound of subfraction F2.4 could decrease the virulence and pathogenesis of *C. neoformans*. Interestingly, the presence of a capsule in *C. neoformans *interferes with the inhibition of pigmentation by subfraction F2.4. Furthermore, we did not observe interference in melanin synthesis of B_16_F_10 _cells in concentrations ≤100 μg/ml, which may imply a selective action of the polymeric tannin from barbatimão on *C. neoformans*.

The cytotoxic effect of the subfraction F2.4 from *S. adstringens *was previously evaluated against mammalian cells (Vero, macrophage J774G8 and red blood cells) by Ishida et al [13]. The cytotoxic concentration of 50% (CC_50_) of the subfraction F2.4 for those cells was higher than 100 μg/ml [13]. It represented a selective index (SI = CC_50_/MIC) to *C. neoformans *of 33 and 20 to Vero and J774G8 cells, respectively. In addition, no haemolytic effect was observed in concentrations lower than 1000 μg/ml of the subfraction F2.4 [13]. In contrast, polyene agents (such as amphotericin B) show a larger haemolytic pattern when compared to the crude extract of *S. adstringens *[11].

## Conclusion

In accordance with previous studies reported on *C. albicans *[13], the hexameric compound extracted from barbatimão works on *C. neoformans *as a cytostatic antifungal agent. It displays an important biological activity against *C. neoformans *interfering with: cellular homeostasis, yeast growth, polysaccharide capsule formation and fungal pigmentation. In addition, the subfraction F2.4 presented low citotoxicity against mammalian cells [13].

These data together show the potential of the hexameric compound to start the development of a new antifungal agent to treat not only cryptococcosis or candidiasis but it could also be used against other types of mycosis.

## Competing interests

The authors declare that they have no competing interests.

## Authors' contributions

KI carried out all experiments and data analysis. KI, SR and CVN participated in the design of the study and in the manuscript writing. JCPM collaborated in the extraction and identification of the hexameric compound of the subfraction F2.4 from *S. adstringens*. All authors have read and approved the final manuscript.
